# Cavopulmonary support with a modified cannulation technique in a failing Fontan patient

**DOI:** 10.1093/icvts/ivac090

**Published:** 2022-04-08

**Authors:** Sebastian G Michel, Ares K Menon, Nikolaus A Haas, Jürgen Hörer

**Affiliations:** 1 Division of Congenital and Pediatric Heart Surgery, Department of Cardiac Surgery, Ludwig Maximilian University Munich, Munich, Germany; 2 Department of Congenital and Pediatric Heart Surgery, German Heart Center Munich, Technical University of Munich, Munich, Germany; 3 Berlin Heart GmbH, Berlin, Germany; 4 Department of Pediatric Cardiology and Intensive Care, Ludwig Maximilian University Munich, Germany

**Keywords:** Failing Fontan, Ventricular assist device, Single ventricle, Congenital heart disease

## Abstract

Failing Fontan patients present a unique challenge for mechanical circulatory support. We report on a 17-year-old patient with Fontan failure and preserved ventricular function who underwent mechanical cavopulmonary support using a novel cannulation technique.

## INTRODUCTION

The failure of a Fontan circulation often leads to venous and lymphatic congestion, ascites and protein-losing enteropathy (PLE). Preserved ventricular function in failing Fontan patients is an independent risk factor for mortality after heart transplantation. These patients have higher PVR, higher central venous pressures and more structural liver changes [1].

In this subset of patients, cavopulmonary support is required to improve organ function. This concept was first successfully described by Prêtre *et al.* [2] who took down the cavopulmonary connections and shaped a new capacity chamber with multiple Dacron patches where the superior and inferior vena cava could drain into ventricular assist device (VAD) inflow. The outflow cannula was connected to the pulmonary artery. This was a huge and technically demanding operation. In recent years, the European EXCOR Paediatric Investigator Group attempted to simplify this procedure by developing a specific ‘Fontan’ cannula, the first use of which is described here.

## DATA AVAILABILITY STATEMENT

The data underlying this article are available in the article.

## ETHICAL STATEMENT

The institutional review board approved this study (code 21-0409).

## CASE REPORT

We report on a 17-year-old man with double-inlet left ventricle and pulmonary stenosis. He had undergone placement of an aorto-pulmonary shunt, partial cavopulmonary connection, thrombectomy of the left pulmonary artery with patch enlargement and extracardiac total cavopulmonary connection with an 18-mm Goretex conduit at the age of 19 months to complete the Fontan palliation. In addition, he had multiple interventions to stent the pulmonary arteries and to fenestrate the Fontan circulation. Nevertheless, he developed PLE and was listed for heart transplantation. He deteriorated clinically, and his functional status gradually disqualified him for heart transplantation. Therefore, we decided to implant a Berlin Heart EXCOR^®^ ventricular assist device in the cavopulmonary circulation as a bridge to candidacy.

Haemodynamic parameters before the procedure were as follows: central venous pressure 20 mmHg, left atrial pressure 10 mmHg and transpulmonary gradient 10 mmHg. The arterial oxygen saturation was 85% with a right-to-left-shunt over the fenestration. The function of the left ventricle was normal. Abdominal sonography showed ascites, enlarged liver veins and liver fibrosis.

**Figure 1:**
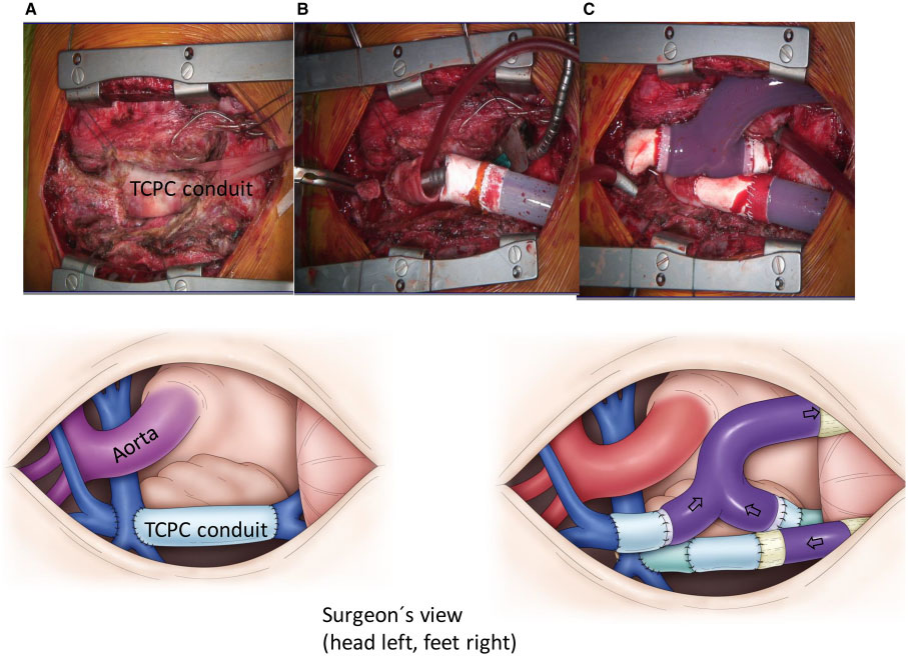
Surgeons view: head left, feet right. (**A**) Fontan patient before VAD implantation: total cavopulmonary connection. (**B**) Total cavopulmonary connection conduit divided and superior part sutured to outflow cannula. (**C**) After implantation of the cavopulmonary assist device: the Y-shaped venous cannula is draining the superior and inferior vena cava (VAD inflow cannula).

As he had 5 previous sternotomies and extensive adhesions were expected, it was decided to cannulate the femoral vessels and the internal jugular vein for full cardiopulmonary bypass without the need for extensive surgical dissection and central cannulation. On pump beating heart, the partial cavopulmonary connection was taken down, and the Fontan fenestration was closed by direct suture. The resulting defect on the right pulmonary artery was repaired with a bovine pericardial patch. The 18-mm total cavopulmonary connection conduit was divided and the superior part was anastomosed to the Goretex graft extension (18 mm) of the VAD outflow cannula. The inferior part was anastomosed to the Goretex graft extension (20 mm) of the inferior part of the novel venous cannula (inflow). The SVC was anastomosed to a 16-mm Goretex graft extension of the superior part of the venous cannula (inflow, Fig. 1). The cannulae were connected to the EXCOR^®^ ventricle (50 ml). Cardiopulmonary bypass time was 180 min. The patient was extubated on postoperative day 2. The PLE symptoms and renal function had improved after 1 month and he was relisted for heart transplant 3 months after VAD implantation. Heart transplant was performed 4.5 months after VAD implantation. He was discharged from the hospital in good clinical condition after a long recovery process 5 months after heart transplant.

## COMMENT

The implantation of a pulsatile sub-pulmonary assist device in failing Fontan patients with a preserved ejection fraction of the systemic ventricle has been described previously by Prêtre *et al.* [2] and Moosmann *et al.* [3]. The concept has been shown to be successful in reducing symptoms of venous and lymphatic congestion; however, until now, it was a very complicated procedure. Using the novel ‘Fontan cannula’, this operation is simplified by reducing the number of necessary anastomoses to only 3. If the function of the systemic ventricle is impaired, this system can be upgraded to a biventricular assist device by cannulating the systemic ventricle or atrium and the ascending aorta and connecting it to a second Berlin Heart^®^ pump. With this type of support, we hope to be able to improve outcomes after heart transplantation for Fontan patients by recovering the function of their secondary organs before transplant.
